# Theorems and Methods of a Complete Q Matrix With Attribute Hierarchies Under Restricted Q-Matrix Design

**DOI:** 10.3389/fpsyg.2018.01413

**Published:** 2018-08-08

**Authors:** Yan Cai, Dongbo Tu, Shuliang Ding

**Affiliations:** ^1^School of Psychology, Jiangxi Normal University, Nanchang, China; ^2^School of Computer and Information Engineering, Jiangxi Normal University, Nanchang, China

**Keywords:** Q matrix, attribute hierarchies, cognitive diagnosis, cognitive diagnostic models, Q matrix design

## Abstract

The design of test Q matrix can directly influence the classification accuracy of a cognitive diagnostic assessment. In this paper, we focus on Q matrix design when attribute hierarchies are known prior to test development. A complete Q matrix design is proposed and theorems are presented to demonstrate that it is a necessary and sufficient condition to guarantee the identifiability of ideal response patterns. A simulation study is also conducted to detect the effects of the proposed design on a family of conjunctive diagnostic models. The results revealed that the proposed Q matrix design is the key condition for guaranteeing classification accuracy. When only one type of item pattern in R matrix is missing from the associated test Q matrix, the related attribute-wise agreement rate will decrease dramatically. When the entire R matrix is missing, both the pattern-wise and attribute-wise agreement rates will decrease sharply. This indicates that the proposed procedures for complete Q matrix design with attribute hierarchies can serve as guidelines for test blueprint development prior to item writing in a cognitive diagnostic assessment.

## Introduction

The purpose of a diagnostic assessment is to detect the presence or absence of multiple fine-grained skills based on the observed response data to a set of test questions. Motivated by the No Child Left Behind Act of 2001 (Law, 2002), most developments and applications of diagnostic assessments have occurred in educational contexts, in which the assessments aim to provide students with information regarding whether or not they have mastered each skill in a group of specific skills, which are often generically referred to as attributes. Attributes may function independently (Tatsuoka and Boodoo, 2000), or they can be hierarchically related, meaning the mastery of a certain attribute is a prerequisite to the mastery of another one, therefore dependent (Vosniadou and Brewer, 1992; Kuhn, 2001). The Q matrix, which specifies the attributes measured by each test question, is an important element in a diagnostic assessment, because it is the foundation for a group of statistical models with different assumptions regarding how attributes influence test performance. These models are typically referred to as cognitive diagnostic models (CDMs) or diagnostic classification models (DCM). Such models include the rule space model (RSM; Tatsuoka, 1985), attribute hierarchy methods (AHM; Leighton et al., 2004) and Deterministic Input, Noisy “And” gate (DINA; Junker and Sijtsma, 2001) etc.

The test Q matrix is a linkage between test items and measured attributes, the element *q*_*ij*_ = 1 or 0 indicates that the *j*_*th*_ attribute is or not is measured by the *i*_*th*_ item respectively. The design of a Q matrix plays an important role in a cognitive diagnostic assessment, because it can directly influence the classification accuracy of a CDM. When attributes are independent, the Q matrix design has been investigated from both theoretical and empirical aspects in the literature. One common conclusion from previous studies is that it is important for a Q matrix to contain items that only measure a single attribute, implying that it contains an identity matrix as a sub-matrix. Such a Q matrix was first defined as a complete Q matrix by Chiu et al. (2009), and was later shown to be an important condition for guaranteeing model identifiability for a family of restricted latent class models (Chen et al., 2015; Xu and Zhang, 2016; Xu, 2017) and a condition guaranteeing a consistent nonparametric estimator (Wang and Douglas, 2015). The completeness of Q matrix was also empirically shown to increase classification accuracy according to several simulation studies (e.g., DeCarlo, 2011; Madison and Bradshaw, 2015).

When attributes are hierarchically related to each other, there are two different opinions regarding the Q matrix design. On one hand, several studies have assumed that the item-attribute structure does not necessarily follow the specified attribute hierarchy, and have utilized the unstructured/independent Q matrix design (De La Torre et al., 2010; Templin and Bradshaw, 2014; Liu et al., 2016). Such an assumption is convenient when the test Q matrix is developed following test administration or when the item can be assumed to measure the higher level attribute without measuring the lower level attribute. In such case, the complete test Q matrix can be obtained by including items that measure each attribute individually, and the importance of such a design is addressed above.

On the other hand, another group of researchers believe the items represented by the test Q matrix should reflect the specified attribute hierarchy, because they represent the attribute blueprint or cognitive specifications for test construction (e.g., Leighton et al., 2004; Tatsuoka, 2009). The Q matrix design under this assumption implies that if an item measures one attribute then it should also measure all of its prerequisites. In other words, there should be less than or equal to (2^*K*^ −1) types (*K* is the number of attributes) of item attribute profiles, where all q-vectors not matching the attribute hierarchy are deleted from the Q-matrix (Tatsuoka, 1990, 2009; Leighton et al., 2004). For example, if attribute A1 is the prerequisite of attribute A2, then the item q-vector (01) should be deleted from the Q-matrix. Hereafter, such a Q matrix is referred to as a restricted Q matrix which does not necessarily contain R matrix. Many real examples of restricted Q matrices can in diagnosis assessments from fraction subtraction (Tatsuoka, 1990; De La Torre, 2011; de la Torre et al., 2016;), mathematicals learning (Tatsuoka, 1990; Leighton and Gierl, 2007), critical reading (Wang and Gierl, 2011), syllogistic reasoning (Leighton et al., 2004) etc. If a diagnostic assessment is developed based on a restricted *Q* matrix, then a natural question is how to ensure its identity. This was the main motivation for our study.

This paper investigates the completeness of restricted Q matrix when the attribute hierarchy is specified. In restricted Q matrix design, including an identity matrix is not feasible because the items measuring the higher level attributes will also measure those in the lower level when the attributes are dependent. This means that the condition of completeness in unstructured Q matrix design cannot be satisfied, because the Q matrix cannot contain an identity matrix. Therefore, it is important to investigate the completeness condition for restricted Q matrix design to guarantee accurate classification results.

In this paper, we define a complete restricted Q matrix design based on R matrix and discuss its corresponding statistical properties. We demonstrate that the completeness of the restricted Q matrix is a key condition to guarantee classification accuracy when the analysis is combined with a family of conjunctive CDMs. Simulation studies also reveal the importance of R matrix in classification accuracy. The proposed design is easy to implement and can serve as the blueprint for a cognitive diagnostic assessment prior to item writing. The remainder of this paper is organized as follows. Section 2 outlines useful elements in a cognitive diagnostic assessment. Section 3 introduces several important incidence matrices and discusses their corresponding statistical properties. This is followed by the definition of a complete restricted Q matrix and our main theorems regarding its statistical properties in Section The complete restricted Q matrix design. The procedures for constructing a complete Q matrix are introduced in Section The restricted Q matrix design. The importance of such a complete Q matrix design for the classification accuracy of a family of conjunctive models is illustrated through several simulation studies in Section Numerical Examples. Experimental result are limited to the simulation study.

## Attribute hierarchies and conjunctive CDMs

### Attribute hierarchies

**Figure 1:**
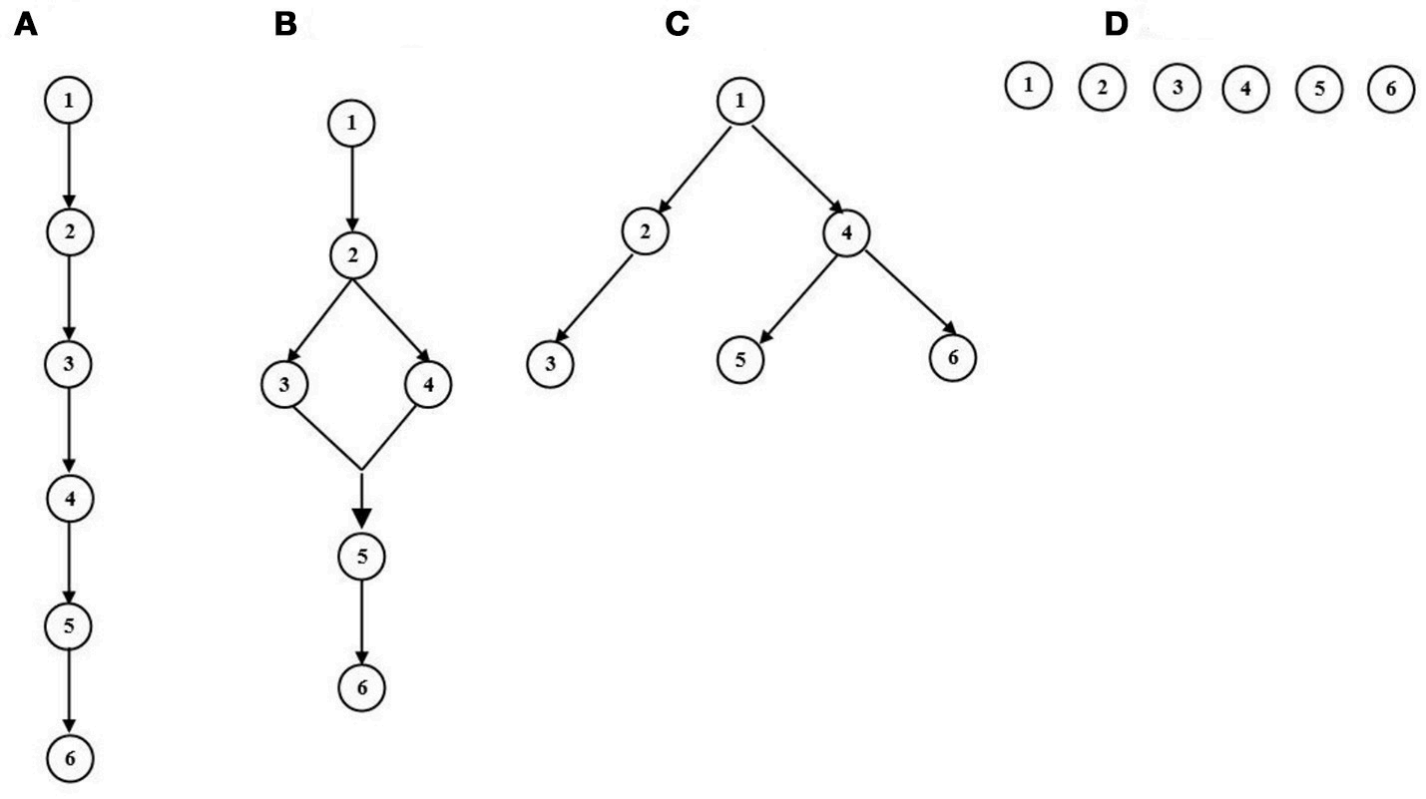
Four types of Attribute Structures. **(A)** Linear. **(B)** Convergent. **(C)** Divergent. **(D)** Independent.

The terminology of attributes was first proposed by Tatsuoka (1990) as “production rules, procedural operations, item types, or more generally any cognitive tasks.” In educational context, this term generally refers to any knowledge or cognitive processing skills required to solve test problems (e.g., Leighton et al., 2004; Tatsuoka, 2009). Attributes can be identified and studied using methods from cognitive psychology, such as item reviews and protocol analysis (Leighton et al., 2004). In a diagnostic assessment, the attribute profile for each subject is denoted by a vector of binary latent variables representing mastery of a finite set of attributes. Suppose there are N examinees and K attributes, we define the *i*_th_ examinee's attribute profile as $\alpha^{i}=(\alpha_{1}^{i},\alpha_{2}^{i},\cdots \;,\alpha_{K}^{i})\prime$, where $\alpha_{k}^{i}\in \left\{0,1\right\}$ to indicate the absence or presence of the *k*_*th*_ attribute for the *i*_*th*_ examinee. The attribute hierarchies refer to situations in which the mastery of a certain attribute is a prerequisite to the mastery of another attribute. Figure 1 presents four types of attribute structures. The linear attribute hierarchy (Figure 1A) requires all attributes to be ordered sequentially, and implies that if attribute 1 is not present, then all following attributes will not be present. The convergent structure (Figure 1B), represents a hierarchy with a convergence branch where two different paths maybe traced from attribute 1 to attribute 3 and 4. Note that in this structure, one attribute can be a prerequisite of multiple different attributes and an attribute can have many different prerequisites. The divergent attribute hierarchy (Figure 1C), refers to different distinct tracks originating from the same single attribute. The independent structure (Figure 1D) can be viewed as a specific case of attribute hierarchy. Note that independence in this sense is not the same as statistical independence. Although no attribute is a prerequisite for another, the indicators of attribute mastery may be correlated.

### Conjunctive cognitive diagnostic models

A rich development of CDMs has occurred over the past decade. Traditional categories for CDMs are based on different assumptions regarding how attributes influence test performance. Most recently, several general models based on different link functions (e.g., Davier, 2008; Henson et al., 2009; De La Torre, 2011) have been developed to include many reduced CDMs. In this study, we focus on the restricted Q matrix design and combine with a conjunctive CDM to perform classification. Conjunctive CDMs assume that all attributes required by an item must be mastered to have high chance to provide the correct answer, which is a reasonable model for classification under certain attribute hierarchies. The combination of Q matrix and CDMs can be found in the application of a math test. For example, researchers have suggested that mathematical concepts are not independent segments, and there are learning sequences within the curriculum that fit the schema-constructing process of learners, which implies certain hierarchical attribute structures (Clements and Sarama, 2004). Usually, in math test, such a set of hierarchical skills are all required to perform well on given item (e.g., Tatsuoka, 1990), and an incorrect answer is highly likely to be provided even if a student is missing only one of the required attributes.

The rule space model (RSM; Tatsuoka, 1985) and attribute hierarchy methods (AHM; Leighton et al., 2004), as well as a family of restricted latent class models, including Deterministic Input, Noisy “And” gate model (DINA; Junker and Sijtsma, 2001), Noisy Input, Deterministic “And” gate model (NIDA; Maris, 1999), and Reparameterized Unified Model (Reduced RUM; Hartz and Roussos, 2008) have conjunctive assumptions. All these conjunctive models rely on the ideal response pattern, which indicates whether or not a subject with a specific attribute profile has mastered all the required attributes for each item, to determine the specific model structure. If we define the column as the item and the row as the attribute in the Q matrix, the ideal response pattern resulting from an attribute pattern **α** = (α_1_, α_2_, ⋯, α_*K*_)′ to a *K* × *J* Q matrix with elements *q*_*kj*_ can be defined as

$$\eta \left(Q,\alpha \right)=\alpha ◦Q={\left(\eta_{1}\left(Q,\alpha \right),\eta_{2}\left(Q,\alpha \right),\ldots ,\eta_{J}\left(Q,\alpha \right)\right)}^{\prime },$$

where $\eta_{j}\left(Q,\alpha \right)=\prod_{k=1}^{K}\alpha_{k}^{q_{kj}}$.

The RSM and AHM perform classification based on the distance between their observed response pattern and the ideal response pattern. The RSM model first maps the observed and ideal response patterns into a two-dimensional space, then considers the points corresponding to the ideal response patterns as the kernels for each class. The remaining points corresponding to the observed response patterns are clustered into classes. The AHM model first treats each observed response pattern as a deviation from the ideal response patterns, then calculates the probabilities of deviations between the observed response pattern and each of the ideal response patterns. Finally, it classifies the examinee with the attribute profile that resulted in the largest deviation probability. Conjunctive restricted latent class models, such as the DINA, NIDA and reduced RUM models, define the probability of a correct response under the conjunctive assumption, but allow for slips and guesses in a manner that distinguishes the models from one another. For example, the DINA model is the simplest conjunctive model, where the item response function is entirely determined by **η**_*j*_(*Q*, **α**). Therefore, there are only two types of correct response probabilities for each item under the DINA model in the item response function:

$$\begin{array}{l} P\left(X_{ij}=1|\alpha \right)=\{\begin{array}{cc} 1-s_{j}, & \text{if}\;\eta_{j}\left(Q,\alpha \right)=1,\\ g_{j}, & if\;\eta_{j}\left(Q,\alpha \right)=0. \end{array} \end{array}$$

In contrast to the DINA model, the slipping and guessing parameters for the NIDA model are defined based on attribute levels. Define*H*_*j*_ = {*k*|*q*_*kj*_ = 1}. Then, for the NIDA model,

$$\begin{array}{l} P\left(X_{ij}=1|\alpha \right)=\{\begin{array}{ll} \prod_{k\in H_{j}}\left(1-s_{k}\right), & \text{if}\;\eta_{j}\left(Q,\alpha \right)=1\\ \prod_{k\in H_{j}}{\left(1-s_{k}\right)}^{\alpha_{k}}g_{k}^{1-\alpha_{k}}, & \text{if}\;\eta_{j}\left(Q,\alpha \right)=0. \end{array} \end{array}$$

The reduced RUM model can be generalized from the NIDA model with an item response function defined as

$$\begin{array}{l} P\left(Y_{ij}=1|\alpha \right)=\{\begin{array}{ll} \pi_{j}, & \text{if}\;\eta_{j}\left(Q,\alpha \right)=1\\ \pi_{j}{\prod_{k\in H_{j}}\left(r_{jk}^{*}\right)}^{1-\alpha_{k}} & \text{if}\;\eta_{j}\left(Q,\alpha \right)=0. \end{array} \end{array}$$

## Preparations for Q matrix design

In this section, five types of matrices are introduced to describe three types of relationships: item vs. attribute, attribute vs. attribute, and examinee vs. attribute. The statistical properties associated with these matrices are discussed to provide a foundation for the proposed Q matrix in the next section. For ease of presentation, we assume that there are K attributes associated with J items and define columns as items and rows as attributes for the incidence matrix describing the relationships between items and attributes.

### The incidence Q matrix and test Q matrix

The incidence Q matrix is defined as a *K*×(2^*K*^−1) matrix that contains items that probe all combinations of attributes when they are independent (Leighton et al., 2004). For example, when *K* = 4, the incidence Q matrix documents a total of 15(= 2^4^−1) possible item types, excluding the type with all elements equal to zero. The test Q matrix is a *K*×*J* matrix, indicating which item measures which attribute in the designed test. Here, J is the number of test questions and it can be less than, equal to, or greater than 2^*K*^−1.

### The reachability matrix

**Figure 2:**
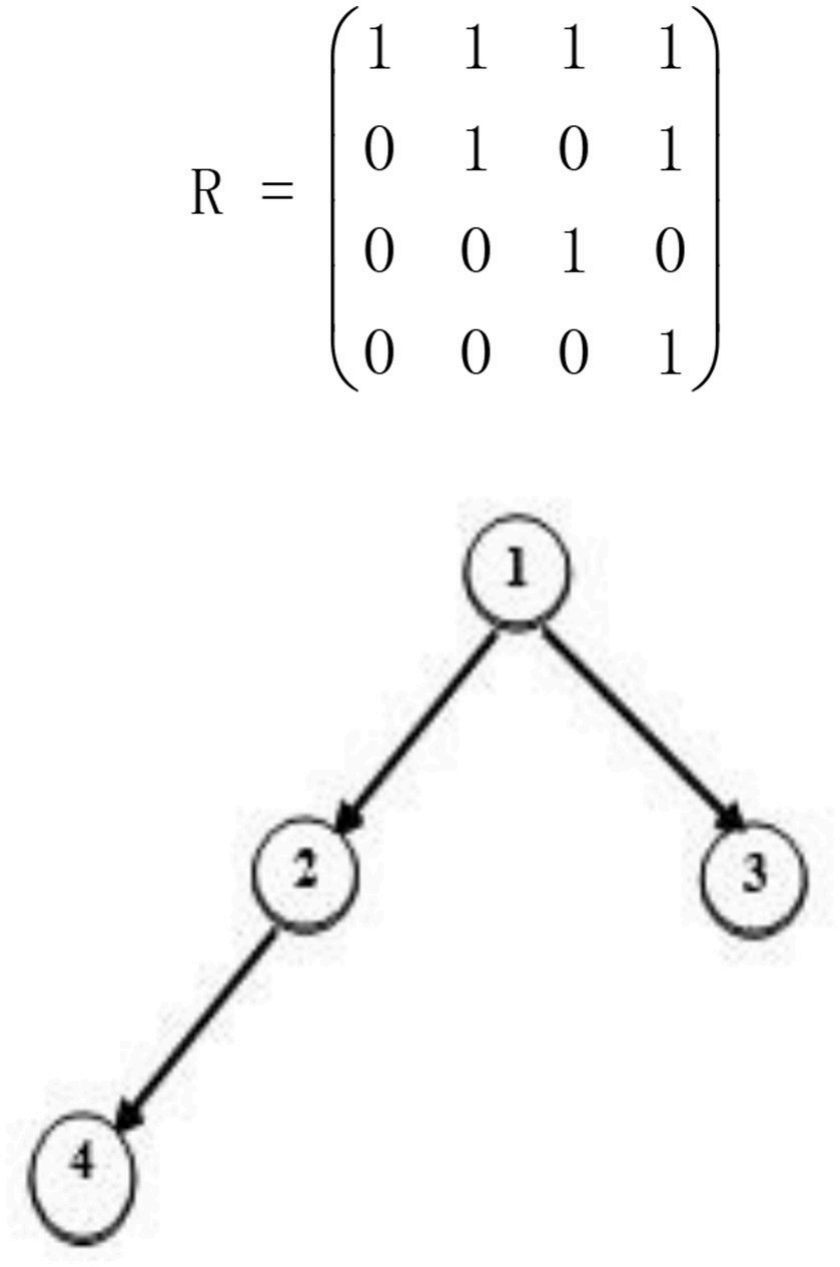
A divergent attribute hierarchy.

The reachability matrix (R matrix; Tatsuoka, 1986) is a *K*×*K* matrix that represents the direct and indirect relationships between attributes. The *j*_*th*_ element of the *i*_*th*_ row in the matrix represents whether attribute *i* is a direct or indirect prerequisite for attribute *j*. Therefore, the *i*_*th*_ row of the R matrix specifies all the attributes, including the *i*_*th*_ attribute, for which the *i*_*th*_ attribute is a direct or indirect prerequisite. Based on the study by Tatsuoka (1986, 2009), the R matrix can be calculated from (**A** + **I**)^*n*^, where n is the integer required for R to reach invariance, A is the adjacency matrix, which is a *K*×*K* binary matrix that specifies the direct relationships between attributes, and **I** is an identity matrix. The R matrix for the divergent attribute hierarchy is presented in Figure 2.

For this R matrix, row one indicates that attribute 1 is a prerequisite to all attributes and row two indicates that attribute 2 is a prerequisite for attribute 2 and 4. The rest of the matrix can be interpreted in the same manner. Let *r*_*ij*_ denote the (i, j) elements in the R matrix, where the *j*_*th*_ column of the R matrix is denoted ${\text{r}}_{j}={\left(r_{1j},\cdots \;,r_{Kj}\right)}^{\prime }$. It is clear that

$$\begin{array}{l} r_{kk}=1 \end{array}$$

for *k* = 1, 2, ⋯ , *K*. To simplify our notation, we always label the attributes from the lowest level to the highest level utilizing an ascending sequence ranging from 1 to K. Therefore, the R matrix is an upper-triangular matrix in our scheme.

### Reduced Q matrix

The Reduced Q matrix (Leighton et al., 2004), denoted *Q*^*r*^, is obtained by removing the items (columns) that do not satisfy the specified hierarchical structure from the incidence Q matrix. In other words, the *Q*r matrix contains all possible item types under the specified hierarchical structure. The *Q*^*r*^ matrix for Figure 2 can be written as follows:

$$\begin{array}{l} Q^{r}=\left(\begin{array}{c} 111111\\ 010111\\ 001011\\ 000101 \end{array}\right) \end{array}$$

Note that there were 2^4^−1 = 15 columns in the incidence Q matrix when the attributes were independent. By removing the seven columns that represented the seven item types not satisfying the attribute hierarchy in Figure 2, we can derive the above *Q*^*r*^ matrix.

### The permissible attribute profile matrix

The previously presented matrices defined two types of relationships: relationships between attributes and the relationships between items and attributes. The next type of matrix defines the relationships between examinees and attributes. We first define a permissible attribute pattern as an attribute pattern that satisfies the specified attribute hierarchy. The permissible attribute profile matrix M is a matrix that is formed with all permissible attribute patterns as columns. The M matrix can be obtained by adding a column vector of zeros to the Qr matrix. Specifically, $M=\left(0_{K},Q^{r}\right)$, where $0_{K}={\left(0,0,\;\cdots \;,0\right)}^{\prime }$. The M matrix defined based on Figure 2 is

$$\begin{array}{l} M=\left(\begin{array}{c} 0111111\\ 0010111\\ 0001011\\ 0000101 \end{array}\right). \end{array}$$

Note that although the M matrix can be constructed from the *Q*^*r*^ matrix, these two matrices represent different explanations. Each column of the M matrix represents a permissible attribute pattern, whereas each column of the *Q*^*r*^ matrix represents an item that satisfies the specified attribute hierarchy (correspondingly, a permissible item type). M defines a permissible attribute profile space. We utilize the notation **α** ∈ *M* to indicate that α belongs to one of the column vectors of M.

### Propositions

In this subsection, several propositions that serve as foundations for the main theorems in the next section are introduced.

#### Proposition 1

Each column of the R matrix represents an item that satisfies the specified hierarchical structure.

Proposition 1 is a restatement of Lemma 2 from the paper by Yang et al. (2008). Its proof is presented in the Appendix (Supplementary Material). This proposition implies that the R matrix can still be reviewed as an incidence matrix that denotes the relationships between items and attributes, although it was originally defined as the relationships between attributes. Leighton et al. (2004) stated that “the R matrix is used to create a subset of items that is conditioned on the structure of the attribute hierarchy.”

The next proposition reveals the relationships between the R matrix and *Q*^*r*^ matrix.

#### Proposition 2

The R matrix is a sub-matrix of the *Q*^*r*^ matrix. That is to say, the R matrix corresponding to a specified attribute hierarchy can be obtained by removing certain columns from the *Q*^*r*^ matrix.

Proof. Proposition 2 can be easily proved by Proposition 1 and the definition of the *Q*^*r*^ matrix from Section The incidence Q matrix and test Q matrix.

The next proposition depends on the definition of Boolean operations (Davey and Priestley, 1990; Tatsuoka, 1991), where the multiplications and additions are defined as follows:

$$\begin{array}{l} \begin{array}{ccc} 1\times 1=1,\; & 1\times 0=0\times 1=0,\; & 0\times 0=0,\\ 1+1=1,\; & 1+0=0+1=1,\; & 0+0=0.\\ \; \end{array} \end{array}$$

We further define the addition and multiplication of two vectors by performing element-wise addition and multiplication of the corresponding elements in the vectors. For example,

$$\begin{array}{l} {\left(1,0,0\right)}^{\prime }\times {\left(0,0,1\right)}^{\prime }={\left(1\times 0,0\times 0,0\times 1\right)}^{\prime }={\left(0,0,0\right)}^{\prime }\\ {\left(1,0,0\right)}^{\prime }+{\left(0,0,1\right)}^{\prime }={\left(1+0,0+0,0+1\right)}^{\prime }={\left(1,0,1\right)}^{\prime }. \end{array}$$

Hereafter, the additions and multiplications for any vectors in different incidence matrices (R, Q^*r*^, and M) follow the above definitions and rules.

#### Proposition 3

Let S represent a hierarchical structure for K attributes, where *R* = (**r**_1_, ⋯ , **r**_*K*_) is the corresponding reachability matrix and **r**_*i*_, *i* = 1, 2, ⋯ , *K* represents the *i*_*th*_ column of the R matrix. Suppose that ${\text{q}}^{r}={\left(b_{1},b_{2},\cdots \;,b_{K}\right)}^{\prime }$ with *b*_*i*_ ∈ {0, 1} is a column of the *Q*^*r*^ matrix. Then, ${\text{q}}^{r}=b_{1}\times {\text{r}}_{1}+b_{2}\times {\text{r}}_{2}+\cdots +b_{k}{\text{r}}_{k}=\overset{K}{\underset{k=1}{\sum }}b_{k}{\text{r}}_{k}$.

Proposition 3 is a restatement of Theorem 1 from the paper by Yang et al. (2008). Its proof is presented in the Appendix (Supplementary Material). Let **q**^*r*^ = (1, 0, 1, 0)′ be the third column of *Q*^*r*^ for the attribute hierarchy specified in Figure 2. Then,

$$\begin{array}{l} {\text{q}}^{r}={\left(1,0,1,0\right)}^{\prime }=1\times {\left(1,0,0,0\right)}^{\prime }+0\times {\left(1,1,0,0\right)}^{\prime }\\ +1\times {\left(1,0,1,0\right)}^{\prime }+0\times {\left(1,1,0,1\right)}^{\prime }\\ =b_{1}\times {\text{r}}_{1}+b_{2}\times {\text{r}}_{2}+b_{3}\times {\text{r}}_{3}+b_{4}\times {\text{r}}_{4} \end{array}$$

The above proposition and example indicate that any column of *Q*^*r*^, denoted **q**r, can be written as a positive linear combination of one or more (≥1) columns from the corresponding R matrix, ${\left\{{\text{r}}_{i}\right\}}_{i=1}^{l}$ (note that we reorder these columns from 1 to *l*, not necessarily corresponding to their positions in the R matrix. Specifically, **q^r^**+**r_i_**=**q^r^**, *i* = 1, 2, ⋯ , *l*, meaning any attributes measured by **r**_*i*_ are a subset of the attributes measured by **q**^r^, which can be written as

$\left\{m|r_{mi}=1\right\}\subset \left\{m|q_{m}^{r}=1\right\}$.

## The complete restricted Q matrix design

From our review in Section Attribute Hierarchies and Conjunctive CDMs, we determined that ideal response patterns play an important role in conjunctive CDM frameworks. It is important for the designed Q matrix to identify different ideal response patterns. Any two different attribute patterns **α**^1^ ≠ **α**^2^ will result in different ideal response patterns **η**(*Q*, **α**^1^) ≠ **η**(*Q*, **α**^2^). To achieve this goal, we define a complete Q matrix and theoretically demonstrate that this is a necessary and sufficient condition to guarantee the identifiability of an ideal response pattern. Completeness is discussed for the case of a restricted Q matrix design and we formally introduce the definition of a restricted Q matrix design below.

**Definition 1**. A restricted Q matrix is defined such that any item in it satisfies the specified attribute hierarchy. In other words, each column of the Q matrix is one of the columns of the *Q*^*r*^ matrix.

For the divergent attribute hierarchy specified in Figure 2, according Definition 1, Q_1_ is a restricted Q matrix and Q_2_ is an unrestricted Q matrix because items two through four violate the specified attribute structure.

$$Q_{1}=\left(\begin{array}{ccccc} 1 & 1 & 1 & 1 & 1\\ 0 & 1 & 0 & 1 & 1\\ 0 & 0 & 1 & 0 & 1\\ 0 & 0 & 0 & 1 & 0 \end{array}\right)\;Q_{2}=\left(\begin{array}{ccccc} 1 & 0 & 0 & 0 & 1\\ 0 & 1 & 0 & 0 & 1\\ 0 & 0 & 1 & 0 & 1\\ 0 & 0 & 0 & 1 & 0 \end{array}\right)$$

### Completeness

**Definition 2**. A restricted Q matrix, denoted *Q*_*c*_, is said to be complete if it satisfies the following condition:

**Condition (I)**. The R matrix is a sub-matrix of the Q matrix, where the restricted Q matrix takes the following form:

$$Q_{c}=\left(RQ_{rest}\right),$$

where *Q*_*rest*_ is formed from the columns *Q*^*r*^.

Remark 1. Definition 2 is equivalent to Definition 1 in the paper by Xu and Zhang (2016) when attributes are independent.

Continuing the example from Figure 2, under the restricted Q matrix design, the Q_1_ matrix defined above is complete, where the Q_3_ matrix defined below is incomplete because the fourth column of the corresponding R matrix (1, 1, 0, 1)′ is not contained in Q_3_. If Q_3_ is utilized as the test Q matrix, then the examinee with the attribute profile **α**^1^ = (1, 1, 0, 1)′ cannot be distinguished from the examinee with the attribute profile **α**^2^ = (1, 1, 0, 0)′ because the two have the same ideal response pattern (1, 1, 0, 1, 0).

$$\begin{array}{l} Q_{3}=\left(\begin{array}{ccccc} 1 & 1 & 1 & 1 & 1\\ 0 & 1 & 0 & 1 & 1\\ 0 & 0 & 1 & 0 & 1\\ 0 & 0 & 0 & 0 & 0 \end{array}\right) \end{array}$$

### Properties of R matrix

In this section, we provide various properties to prove the completeness of Q matrix defined above.

Lemma 1. Suppose that *r*_*j*_(*j* = 1, ⋯ , *K*) is the *j*_*th*_ column vector of the R matrix. Then, it holds that*r*_*j*_◦*R* = *r*_*j*_.

Lemma 1 implies that the ideal response pattern of an individual with a knowledge state *r*_*j*_ in R matrix is still *r*_*j*_. This result can be easily obtained from the definition of α◦*Q*. According to the definition of an R matrix, the following Lemmas 2 and 3 can also be easily obtained.

Lemma 2. Suppose that *r*_*j*_ is the *j*_*th*_ column vector of the R matrix and that it contains at least two non-zero entries. Then, it satisfies

1. *r*_*jj*_ = 1, and there exists some *i* satisfying *r*_*ij*_ = 1, where attribute *i* is a prerequisite of attribute *j*.
2. All the prerequisites of the attribute *i* are the prerequisites of attribute *j*.
3. All the entries in the *r*_*j*_−*r*_*i*_ vector are nonnegative. In this paper, *r*_*i*_ ≤ *r*_*j*_ is utilized to represent this type of relationship.

Lemma 3. Suppose that attribute *i* is the only direct prerequisite of attribute *j*. Then, *r*_*i*_ ≤ *r*_*j*_ and ${\left(r_{j}-r_{i}\right)}^{\prime }\left(r_{j}-r_{i}\right)=1$, which indicates that *r*_*i*_ and *r*_*j*_ only differ in their *j*_*th*_ entry and *r*_*ij*_ = 0, *r*_*jj*_ = 1.

Denote $Q^{r}=\left(R,Q_{rest}^{r}\right)$ and let $R_{qj}^{*}$ denote an altered matrix where the *j*_*th*_ column of the R matrix is replaced by some *q* column vector from the $Q_{rest}^{r}$ matrix.

Lemma 3. Suppose that attribute *i* is the only direct prerequisite of attribute *j*. Then, *r*_*i*_ and *r*_*j*_ satisfy $r_{i}◦R_{qj}^{*}=r_{j}◦R_{qj}^{*}$.

According to Lemma 1, it holds that*r*_*j*_◦*R* = *r*_*j*_,*r*_*i*_◦*R* = *r*_*i*_. Because R and $R_{qj}^{*}$ only differ in the *j*_*th*_ column and *r*_*i*_ and *r*_*j*_ only differ in the *j*_*th*_ entry according to Lemma 2, $r_{i}◦R_{qj}^{*}$ and $r_{j}◦R_{qj}^{*}$ could at most differ in the *j*_*th*_ entry, which is determined by *r*_*i*_◦*q* and *r*_*j*_◦*q*, respectively. According to the definition of α◦*Q*, *r*_*i*_◦*q* = *r*_*j*_◦*q* = 0 is satisfied, which proves Lemma 3.

Lemma 4. Suppose that there at least two direct prerequisites of attribute *j*, denoted *i*_1_, *i*_2_, ⋯ , *i*_*k*_(*k* ≥ 2). Then, a vector *p* can be obtained by $p=\overset{K}{\underset{l=1}{\sum }}r_{i_{l}}$, which satisfies $p◦R_{qj}^{*}=r_{j}◦R_{qj}^{*}$.

According Lemma 2, for any *l*, it holds that *r*_*i*_*l*__ ≤ *r*_*j*_. Therefore, *p* ≤ *r*_*j*_. The relationships between attribute *j* and all its prerequisites are reflected in *r*_*i*_*l*__ for all *l*, meaning they are also reflected in *p* and ${\left(r_{j}-p\right)}^{\prime }\left(r_{j}-p\right)=1$. This indicates that *p* only differs from*r*_*j*_ in the *j*_*th*_ entry. According to Lemma 3, it can concluded that $p◦R_{qj}^{*}=r_{j}◦R_{qj}^{*}$. Together, Lemmas 3 and 4 imply that for any *r*_*j*_, one could find another knowledge state α such that their ideal response patterns to $R_{qj}^{*}$ are the same. Additionally, α ≤ *r*_*j*_, and α and *r*_*j*_ only differ in their *j*_*th*_ entry (i.e., *a*_*j*_ = 0, *r*_*jj*_ = 1).

### Main theorems

In this section, we provide theorems to prove that the complete restricted Q matrix defined above can identify any pair of different attribute profiles based on an ideal response pattern.

#### Theorem 1

For any two different attributes profiles **α**^1^, **α**^2^ ∈ *M*, **η**(*R*, **α**^1^) ≠ **η**(*R*, **α**^2^), where **η**(*R*, **α**) = **α**◦*R*.

Theorem 1 implies that the R matrix can be viewed as a complete Q matrix itself. As discussed in Proposition 1, we indicate that any column of the R matrix can be treated as a reasonable item type under the specified attribute hierarchy. This theorem also reveals the importance of including the R matrix in a restricted Q matrix because it is a sufficient condition to identify two distinct ideal response patterns. The next theorem demonstrates that including the R matrix in the restricted Q matrix design is not only a sufficient condition, but also a necessary condition for the identifiability of an ideal response pattern.

#### Theorem 2

Denote the restricted Q matrix design as *Q*_*c*_. For any two different permissible attribute profiles, $\alpha^{1}\neq \alpha^{2}\to \eta \left(Q_{c},\alpha^{1}\right)\neq \eta \left(Q_{c},\alpha^{2}\right)$ if and only if *Q*_*c*_ is complete. That is to say, the R matrix is a sub-matrix of *Q*_*c*_.

When limiting to restricted Q matrix design, proposition 9 in the paper by Heller et al. (2015) is equivalent to the sufficiency of theorem 2. However it does not proof the necessity of identifiability, theorem 2 proved it. When attributes are independent, the R matrix becomes a K × K identity matrix and Theorem 2 follows from Lemma 1 in the paper by Chiu et al. (2009), which indicates that a complete restricted Q matrix must include an identity matrix. The completeness of the Q matrix is a very important condition for model identifiability, particularly for the family of conjunctive CDMs, where ideal response patterns play an important role in the model structure. The results regarding model identifiability for the DINA model (Xu and Zhang, 2016) can be easily generalized for attribute hierarchies by replacing the condition (C1) with the proposed **Condition (I)**. In fact, when item parameters are known for the DINA model, the completeness of the Q matrix is equivalent to model identifiability.

## The restricted Q matrix design

Previous sections illustrated the importance of the R matrix in restricted Q matrix design. In general, a complete restricted Q matrix can be constructed by first creating an R matrix based on specified attribute hierarchy and then selecting a number of columns from the Qr matrix as additional item types for the Q matrix. However, it is difficult to derive the corresponding Qr matrix by removing columns that do not satisfy the specified attribute hierarchy from the incidence Q matrix when K is large. In this section, we introduce the augment algorithm proposed by Ding et al. (2008) and Yang et al. (2008), which can derive a Qr matrix from the corresponding R matrix. The convergence of this algorithm was proved by Proposition 3 in this paper and Theorem 3 in the paper by Yang et al. (2008).

### The augment algorithm for deriving a *Q^*r*^* matrix

The augment algorithm for deriving a *Q*^*r*^ matrix from the corresponding R matrix is presented below.

**Algorithm 1:**
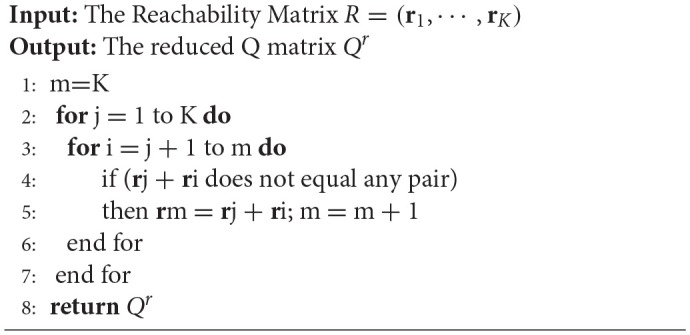
Augment Algorithm

**Figure 3:**
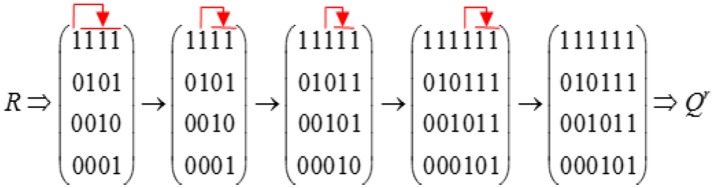
Example of constructing the Qr matrix based on the Augment Algorithm.

Here, we provide an example to demonstrate how to construct a *Q*^*r*^ matrix based on the attribute hierarchy in Figure 2. This process is illustrated in Figure 3. We first obtain the R matrix for this divergent structure, which forms the first four columns of the *Q*^*r*^ matrix. Then, Boolean addition is applied between the first column and each of the remaining three columns. No new item types are produced during this procedure. Then, Boolean addition is applied between the second column and each of the remaining two columns. A new item type (1, 1, 1, 0)′ results from (1, 1, 0, 0)′+(1, 0, 1, 0)′, so we add this item type as a fifth column in the *Q*^*r*^ matrix. Next, we apply Boolean addition between the third column and the fourth and fifth columns. This results in another new item type (1, 1, 1, 1)′, which is added as a sixth column in the *Q*^*r*^ matrix. Finally, we determine that no new item types can be created by applying Boolean addition between the fourth column and the fifth and sixth columns. Therefore, we terminate the searching algorithm.

### Procedures to construct a complete test Q matrix

If a conjunctive model is utilized to analyze data, we provide some suggestions regarding how to perform complete restricted Q matrix design prior to item creation. Suppose that there are K attributes associated with the blueprint for a test with J (≥K) items.

1. Create an R matrix from the corresponding attribute hierarchy.
2. Generate a *Q*^*r*^ matrix by utilizing the augment algorithm.
3. Include the R matrix as the first K columns of the test Q matrix, then randomly select the remaining J–K (if > 0) columns from the columns of the *Q*^*r*^ matrix (this step can be modified to select columns from the *Q*^*r*^ matrix that satisfy the blueprint requirement).

Note that another important feature for Q matrix design is the number of items measuring the same attribute. This feature can be important in terms of model identifiability (Chen et al., 2015; Xu and Zhang, 2016; Xu, 2017). In future studies, we will investigate how to incorporate this feature into restricted Q matrix design.

## Numerical examples

Several numerical examples utilizing five conjunctive CDMs, namely the rule space model (RSM), attribute hierarchical model (AHM), and three conjunctive models (the DINA, NIDA, and reduced RUM model), are presented to demonstrate the important role of the R matrix in complete Q matrix construction and its impact on classification accuracy. These CDMs all depend on ideal response patterns for classification. In each of the following examples, four types of attribute structures, namely linear, convergent, divergent, and independent, involving the K = 6 attributes presented in Figure 1 are considered. The R matrices corresponding to the four structures are provided in the Appendix (Supplementary Material).

### Simulation conditions

Three types of Q matrix design are considered for each of the models. In the first type, the test Q matrix is generated by first including all item types in the corresponding R matrix, except one that is assumed to be missing, and then randomly selecting item types from the remaining columns of the corresponding *Q*^*r*^ matrix. The second type of test Q matrix design is created by randomly choosing columns from$Q_{rest}^{r}$, which is an extreme condition where we assume that the test Q matrix does not contain the entire R matrix. Note that only two types of structure, namely independent and divergent, are considered in this case because there will be no available item types for convergent or linear structures after removing the entire R matrix. The type of design, which is the proposed Q matrix design, is created by first including the entire R matrix and then randomly selecting columns from the corresponding *Q*^*r*^ matrix to fill in the rest of items. The purpose of this test is to compare the classification accuracies of the three types of Q matrix design.

In each of the experimental conditions, to obtain more stable experimental results and decrease the impact of random errors, a sample size of *N* = 1,000 examinees is simulated. Because an attribute profile is a discrete variable, the examinee profiles were simulated based on a uniform distribution formed from all permissible attribute patterns in the different attribute hierarchies. The test length was fixed to 30 items across all conditions. For RSM and AHM, student response vectors were generated based on their ideal response patterns in such a manner that the probability for 1 → 0 and 0 → 1 in each of the ideal response patterns was 0.05. The minimum Mahalanobis distance method and Bayes decision rule proposed by Tatsuoka (2009) were utilized to estimate the attribute profiles for the RSM. For the AHM, the A method proposed by Leighton et al. (2004), was utilized to perform classification. For the three restricted latent class models, we first simulated the item parameters and then generated student responses based on the corresponding item response functions. Specifically, for the DINA model, the slipping parameters *s*_*j*_ and guessing parameters *g*_*j*_ were drawn from a uniform distribution ranging from 0.1 to 0.4, which means both the slipping and guessing parameters fall within the interval between 0.1 and 0.4, which represents average item quality. For the NIDA model, the guessing and slipping parameters for each attribute were simulated. The test Q matrices and examinee attribute profiles were kept constant across the 50 simulations under each experiment condition. The classification accuracies were calculated in terms of pattern-wise agreement rate (PAR) and attribute-wise agreement rate (AAR) to reflect the agreement between estimated attribute profiles and known true attribute profiles based on the average results of the 50 simulations. These two indexes are defined as

$$\begin{array}{l} \text{PAR}=\frac{1}{N}\sum_{i=1}^{N}I\left[\hat{\alpha^{i}}=\alpha^{i}\right]\\ \text{AAR}=\frac{1}{NK}\sum_{i=1}^{N}\sum_{k=1}^{K}I\left[\hat{\alpha_{k}^{i}}=\alpha_{k}^{i}\right]. \end{array}$$

## Results

**Figure 4:**
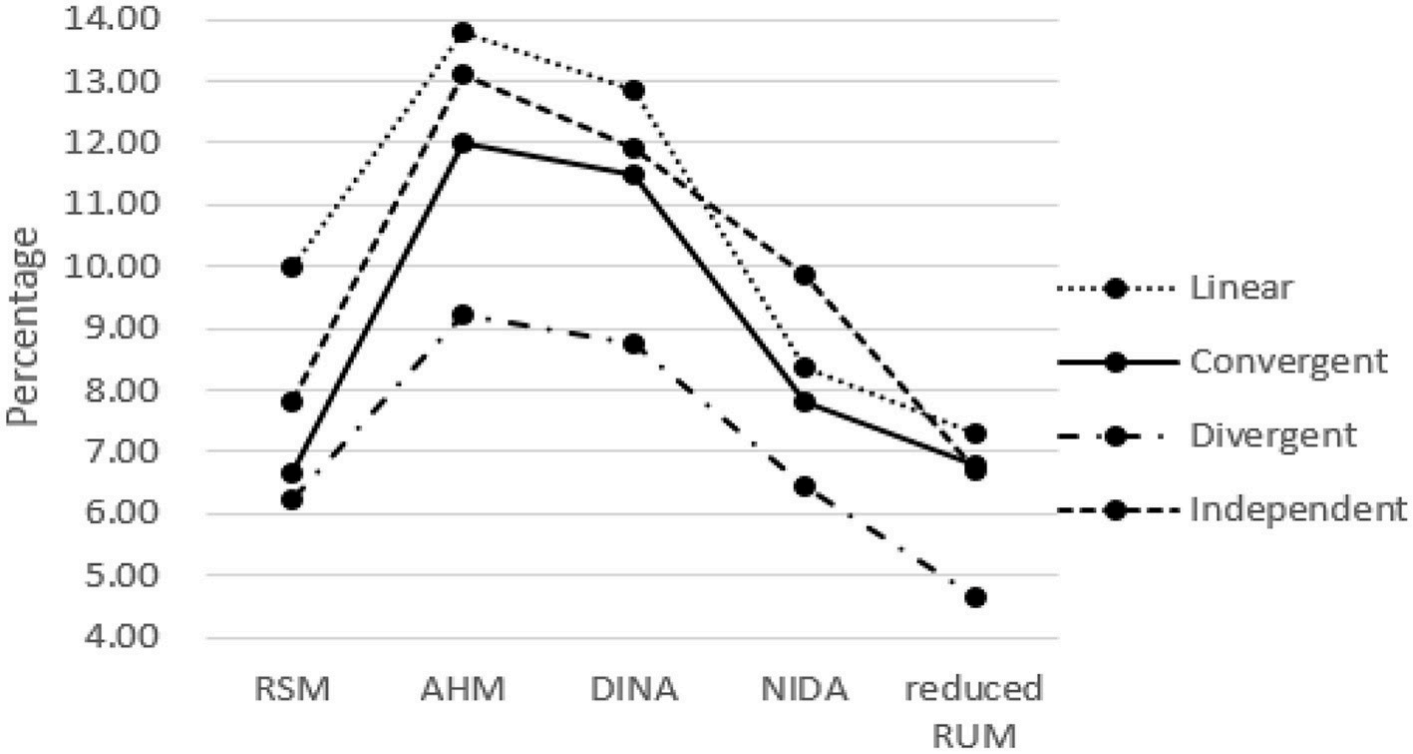
The Average Decrease of AAR when only one item type is missing.

**Figure 5:**
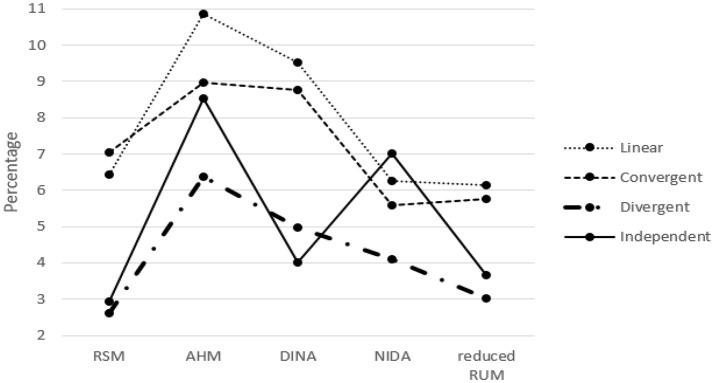
The Average Decrease of PAR when only one item type is missing.

The classification results for the five models when one column of the R matrix is missing from the test Q matrix are listed in Tables 1–5. One can observe consistent results for the five models. When only one column of the R matrix is missing from the test Q matrix, there is a relatively large decrease in the AAR of certain attributes, specifically those that are directly associated with the missing item type. For example, in the linear structure, if the item type (1, 1, 0, 0, 0, 0)′, which measures both attribute one and attribute two, is missing, then the two attribute patterns (1, 1, 0, 0, 0, 0)′ and (1, 0, 0, 0, 0, 0)′ will result in the same ideal response pattern. This leads to a decrease in the AAR for attribute two in this case because attribute two cannot be separated from attribute one during the classification procedure. Another observation is that the overall recovery of attribute patterns, which is represented by PAR, decreases across all conditions when one of the item types is missing from the test Q matrix. To better observe the trends in classification accuracy, we documented the average decreases in AAR and PAR across the six attributes and six missing item types when compared to the classification results from the test Q matrix containing all the item types in the R matrix in Figures 4, 5. The results reveal that the influence of a missing item on classification results are varies under different hierarchical structures. The linear structure has the largest average decrease in AAR across all five models, followed by the independent, convergent, and divergent structures. The trend is similar for PAR, with the exception of the convergent structure producing a larger decrease in PAR than the independent structure in most cases. Furthermore, the influence of a missing item on classification accuracy varies across the different models. The AHM showed the largest decrease in AAR and PAR for each structure, followed by the DINA model. The RSM and NIDA model provided similar performances. The reduced RUM showed the smallest decrease in AAR and PAR across all structures.

**Table 1:** The classification result for RSM when one column of R matrix missing in the test Q matrix.

**Structure**	**The missing item type**	**AAR**						**PAR**
		**A1**	**A2**	**A3**	**A4**	**A5**	**A6**	
Linear	None	0.911	0.903	0.909	0.905	0.9	0.874	0.656
	[100000]'	0.853	0.81	0.838	0.858	0.855	0.84	0.588
	[110000]'	0.901	0.782	0.9	0.931	0.914	0.885	0.577
	[111000]'	0.912	0.917	0.786	0.898	0.922	0.885	0.591
	[111100]'	0.917	0.902	0.906	0.785	0.896	0.882	0.615
	[111110]'	0.906	0.907	0.9	0.91	0.74	0.876	0.575
	[111111]'	0.903	0.904	0.897	0.871	0.826	0.858	0.604
Convergent	None	0.928	0.918	0.869	0.86	0.909	0.896	0.639
	[100000]'	0.871	0.833	0.839	0.814	0.877	0.87	0.556
	[110000]'	0.911	0.809	0.864	0.864	0.926	0.901	0.568
	[111000]'	0.919	0.926	0.799	0.904	0.922	0.898	0.577
	[110100]'	0.916	0.913	0.892	0.789	0.915	0.902	0.565
	[111110]'	0.922	0.927	0.871	0.875	0.836	0.892	0.569
	[111111]'	0.909	0.903	0.844	0.842	0.859	0.876	0.576
Divergent	None	0.963	0.841	0.804	0.895	0.796	0.768	0.526
	[100000]'	0.929	0.862	0.804	0.895	0.812	0.781	0.521
	[110000]'	0.951	0.745	0.784	0.946	0.822	0.811	0.504
	[111000]'	0.956	0.816	0.763	0.918	0.805	0.819	0.504
	[100100]'	0.941	0.865	0.799	0.782	0.766	0.767	0.485
	[100110]'	0.961	0.858	0.795	0.89	0.74	0.822	0.491
	[100101]'	0.953	0.852	0.812	0.891	0.797	0.735	0.494
Independent	None	0.748	0.722	0.738	0.722	0.713	0.738	0.351
	[100000]'	0.645	0.749	0.738	0.718	0.733	0.741	0.316
	[010000]'	0.746	0.655	0.733	0.744	0.722	0.736	0.324
	[001000]'	0.737	0.75	0.648	0.725	0.755	0.742	0.318
	[000100]'	0.738	0.733	0.738	0.655	0.759	0.738	0.328
	[000010]'	0.744	0.737	0.706	0.762	0.65	0.755	0.32
	[000001]'	0.737	0.742	0.73	0.767	0.725	0.659	0.325

**Table 2:** The classification result for AHM when one column of R matrix missing in the test Q matrix.

**Structure**	**The missing item type**	**AAR**						**PAR**
		**A1**	**A2**	**A3**	**A4**	**A5**	**A6**	
Linear	None	0.991	0.985	0.987	0.981	0.988	0.989	0.925
	[100000]'	0.852	0.991	0.993	0.985	0.993	0.995	0.815
	[110000]'	0.99	0.847	0.992	0.989	0.991	0.993	0.816
	[111000]'	0.992	0.986	0.846	0.989	0.996	0.995	0.818
	[111100]'	0.99	0.992	0.99	0.849	0.993	0.993	0.818
	[111110]'	0.993	0.994	0.991	0.989	0.849	0.994	0.82
	[111111]'	0.988	0.989	0.988	0.991	0.992	0.856	0.811
Convergent	None	0.989	0.982	0.98	0.979	0.988	0.985	0.911
	[100000]'	0.875	0.989	0.986	0.982	0.991	0.995	0.824
	[110000]'	0.992	0.867	0.987	0.983	0.991	0.992	0.828
	[111000]'	0.992	0.99	0.852	0.986	0.989	0.995	0.818
	[110100]'	0.992	0.987	0.986	0.847	0.99	0.995	0.814
	[111110]'	0.992	0.989	0.983	0.988	0.87	0.993	0.825
	[111111]'	0.99	0.99	0.981	0.983	0.993	0.877	0.819
Divergent	None	0.992	0.983	0.981	0.987	0.975	0.97	0.899
	[100000]'	0.9	0.982	0.98	0.987	0.979	0.975	0.851
	[110000]'	0.991	0.883	0.976	0.992	0.984	0.981	0.833
	[111000]'	0.993	0.98	0.896	0.988	0.981	0.98	0.836
	[100100]'	0.991	0.988	0.981	0.895	0.975	0.975	0.833
	[100110]'	0.993	0.986	0.981	0.988	0.877	0.982	0.825
	[100101]'	0.991	0.985	0.983	0.986	0.979	0.886	0.833
Independent	None	0.966	0.958	0.961	0.961	0.957	0.961	0.804
	[100000]'	0.831	0.962	0.966	0.96	0.964	0.968	0.724
	[010000]'	0.966	0.842	0.962	0.964	0.961	0.961	0.73
	[001000]'	0.959	0.965	0.815	0.96	0.965	0.96	0.707
	[000100]'	0.963	0.962	0.965	0.833	0.964	0.959	0.722
	[000010]'	0.963	0.96	0.955	0.968	0.828	0.964	0.715
	[000001]'	0.963	0.961	0.959	0.967	0.958	0.83	0.715

**Table 3:** The classification result for DINA when one column of R matrix missing in the test Q matrix.

**Structure**	**The missing item type**	**AAR**						**PAR**
		**A1**	**A2**	**A3**	**A4**	**A5**	**A6**	
Linear	None	0.979	0.979	0.98	0.977	0.978	0.985	0.893
	[100000]'	0.853	0.98	0.985	0.988	0.986	0.99	0.797
	[110000]'	0.983	0.848	0.983	0.985	0.987	0.991	0.799
	[111000]'	0.986	0.981	0.852	0.977	0.989	0.985	0.798
	[111100]'	0.989	0.984	0.981	0.849	0.978	0.988	0.796
	[111110]'	0.987	0.99	0.984	0.981	0.851	0.988	0.801
	[111111]'	0.986	0.989	0.985	0.984	0.982	0.854	0.795
Convergent	None	0.983	0.982	0.974	0.973	0.977	0.978	0.883
	[100000]'	0.873	0.976	0.983	0.975	0.985	0.984	0.797
	[110000]'	0.978	0.865	0.976	0.98	0.982	0.981	0.797
	[111000]'	0.983	0.976	0.846	0.977	0.984	0.986	0.783
	[110100]'	0.989	0.977	0.976	0.852	0.986	0.983	0.794
	[111110]'	0.988	0.982	0.983	0.975	0.868	0.98	0.8
	[111111]'	0.986	0.982	0.979	0.98	0.983	0.874	0.801
Divergent	None	0.976	0.956	0.953	0.954	0.946	0.947	0.794
	[100000]'	0.908	0.959	0.959	0.961	0.952	0.948	0.774
	[110000]'	0.974	0.854	0.951	0.955	0.946	0.956	0.739
	[111000]'	0.979	0.948	0.874	0.959	0.955	0.946	0.74
	[100100]'	0.972	0.947	0.95	0.868	0.943	0.938	0.738
	[100110]'	0.977	0.955	0.963	0.954	0.858	0.954	0.742
	[100101]'	0.977	0.959	0.96	0.955	0.953	0.846	0.732
Independent	None	0.885	0.87	0.88	0.889	0.873	0.869	0.532
	[100000]'	0.771	0.878	0.895	0.885	0.884	0.885	0.507
	[010000]'	0.881	0.744	0.873	0.872	0.883	0.871	0.478
	[001000]'	0.883	0.882	0.761	0.89	0.876	0.872	0.492
	[000100]'	0.881	0.89	0.876	0.755	0.871	0.88	0.491
	[000010]'	0.868	0.874	0.882	0.874	0.754	0.886	0.483
	[000001]'	0.88	0.872	0.883	0.895	0.884	0.765	0.501

**Table 4:** The classification result for NIDA when one column of R matrix missing in the test Q matrix.

**Structure**	**The missing item type**	**AAR**						**PAR**
		**A1**	**A2**	**A3**	**A4**	**A5**	**A6**	
Linear	None	0.989	0.986	0.981	0.975	0.967	0.958	0.865
	[100000]'	0.914	0.99	0.984	0.975	0.97	0.966	0.805
	[110000]'	0.992	0.907	0.984	0.984	0.973	0.968	0.814
	[111000]'	0.995	0.991	0.899	0.982	0.971	0.968	0.813
	[111100]'	0.994	0.988	0.974	0.893	0.969	0.966	0.8
	[111110]'	0.994	0.987	0.984	0.961	0.882	0.961	0.794
	[111111]'	0.996	0.99	0.989	0.978	0.957	0.859	0.788
Convergent	None	0.991	0.984	0.967	0.97	0.967	0.96	0.851
	[100000]'	0.924	0.989	0.974	0.969	0.968	0.968	0.8
	[110000]'	0.992	0.935	0.974	0.976	0.974	0.968	0.828
	[111000]'	0.994	0.985	0.874	0.969	0.970	0.964	0.777
	[110100]'	0.993	0.984	0.967	0.877	0.968	0.964	0.777
	[111110]'	0.994	0.988	0.972	0.973	0.89	0.962	0.803
	[111111]'	0.993	0.99	0.975	0.972	0.96	0.875	0.786
Divergent	None	0.991	0.959	0.946	0.97	0.939	0.941	0.78
	[100000]'	0.927	0.965	0.95	0.975	0.945	0.947	0.76
	[110000]'	0.989	0.899	0.957	0.97	0.935	0.935	0.735
	[111000]'	0.99	0.956	0.872	0.973	0.941	0.941	0.72
	[100100]'	0.989	0.965	0.949	0.923	0.95	0.941	0.761
	[100110]'	0.992	0.961	0.947	0.968	0.867	0.943	0.725
	[100101]'	0.991	0.964	0.952	0.968	0.939	0.871	0.733
Independent	None	0.936	0.926	0.938	0.926	0.924	0.94	0.675
	[100000]'	0.819	0.927	0.918	0.913	0.936	0.927	0.585
	[010000]'	0.933	0.829	0.934	0.931	0.938	0.937	0.621
	[001000]'	0.925	0.937	0.824	0.927	0.922	0.933	0.599
	[000100]'	0.929	0.928	0.93	0.844	0.925	0.925	0.609
	[000010]'	0.919	0.932	0.933	0.922	0.829	0.933	0.601
	[000001]'	0.933	0.928	0.923	0.915	0.935	0.853	0.613

**Table 5:** The classification result for Reduced RUM when one column of R matrix missing in the test Q matrix.

**Structure**	**The missing item type**	**AAR**						**PAR**
		**A1**	**A2**	**A3**	**A4**	**A5**	**A6**	
Linear	None	0.996	0.993	0.991	0.994	0.992	0.99	0.957
	[100000]'	0.935	0.996	0.995	0.995	0.997	0.995	0.914
	[110000]'	0.994	0.931	0.995	0.996	0.995	0.994	0.905
	[111000]'	0.996	0.993	0.933	0.996	0.996	0.993	0.908
	[111100]'	0.996	0.994	0.993	0.935	0.995	0.995	0.909
	[111110]'	0.996	0.995	0.995	0.992	0.925	0.996	0.901
	[111111]'	0.997	0.995	0.996	0.995	0.991	0.859	0.836
Convergent	None	0.993	0.993	0.99	0.991	0.993	0.992	0.952
	[100000]'	0.94	0.996	0.992	0.993	0.996	0.994	0.911
	[110000]'	0.992	0.954	0.994	0.991	0.995	0.992	0.92
	[111000]'	0.994	0.993	0.924	0.99	0.995	0.994	0.895
	[110100]'	0.994	0.993	0.99	0.925	0.995	0.992	0.896
	[111110]'	0.996	0.995	0.995	0.993	0.927	0.992	0.899
	[111111]'	0.993	0.996	0.993	0.995	0.991	0.875	0.845
Divergent	None	0.991	0.982	0.983	0.985	0.977	0.974	0.902
	[100000]'	0.969	0.985	0.984	0.988	0.982	0.981	0.896
	[110000]'	0.991	0.943	0.985	0.987	0.979	0.981	0.879
	[111000]'	0.992	0.982	0.923	0.988	0.978	0.984	0.858
	[100100]'	0.991	0.984	0.982	0.957	0.978	0.983	0.888
	[100110]'	0.991	0.982	0.981	0.985	0.915	0.979	0.851
	[100101]'	0.992	0.985	0.986	0.983	0.982	0.916	0.859
Independent	None	0.945	0.938	0.941	0.941	0.934	0.933	0.72
	[100000]'	0.877	0.94	0.944	0.941	0.936	0.935	0.683
	[010000]'	0.942	0.869	0.933	0.94	0.942	0.939	0.682
	[001000]'	0.941	0.938	0.874	0.941	0.938	0.944	0.687
	[000100]'	0.944	0.944	0.938	0.875	0.942	0.94	0.69
	[000010]'	0.944	0.941	0.945	0.93	0.873	0.93	0.682
	[000001]'	0.94	0.943	0.934	0.942	0.939	0.861	0.677

The classification results of the five models when the entire R matrix is missing from the test Q matrix are listed in Table 6. One can observe a more obvious decrease in AAR and PAR in this extreme case. Specifically, when the entire R matrix is missing from the test Q matrix, compared to the results from the test Q matrix including the entire R matrix, the average decreases in AAR were at least 14.12% for DINA, 13.95% for AHM, 10% for RSM and NIDA, and 7.42% for reduced RUM. Regarding the attribute patterns, the decrease in PAR varied from 16.2 to 44.8% across the five models.

**Table 6:** Classification rates for five conjunctive models when test Q matrix does not contain the entire R matrix.

**Model**	**Structure**	**Test Q Matrix**	**AAR**						**PAR**
			**A1**	**A2**	**A3**	**A4**	**A5**	**A6**	
RSM	Independent	None R	0.663	0.656	0.67	0.677	0.669	0.665	0.237
		All R	0.787	0.794	0.752	0.741	0.763	0.751	0.399
	Divergent	None R	0.85	0.72	0.715	0.765	0.697	0.699	0.347
		All R	0.975	0.826	0.815	0.946	0.819	0.828	0.582
AHM	Independent	None R	0.818	0.83	0.83	0.829	0.833	0.83	0.524
		All R	0.995	0.995	0.995	0.996	0.995	0.996	0.972
	Divergent	None R	0.875	0.82	0.88	0.778	0.872	0.894	0.57
		All R	0.997	0.99	0.993	0.996	0.99	0.99	0.957
DINA	Independent	None R	0.747	0.751	0.75	0.756	0.749	0.743	0.384
		All R	0.955	0.959	0.957	0.958	0.959	0.95	0.766
	Divergent	None R	0.902	0.796	0.869	0.771	0.832	0.824	0.529
		All R	0.994	0.966	0.97	0.978	0.966	0.967	0.856
NIDA	Independent	None R	0.854	0.844	0.84	0.852	0.857	0.841	0.449
		All R	0.981	0.975	0.975	0.978	0.983	0.978	0.877
	Divergent	None R	0.767	0.868	0.91	0.859	0.896	0.905	0.573
		All R	0.997	0.974	0.952	0.983	0.955	0.947	0.823
rRUM	Independent	None R	0.885	0.889	0.882	0.883	0.898	0.887	0.588
		All R	0.976	0.978	0.979	0.979	0.979	0.982	0.879
	Divergent	None R	0.886	0.908	0.941	0.913	0.913	0.944	0.726
		All R	0.999	0.989	0.987	0.995	0.995	0.985	0.939

Additionally, to analyze the effects of the R matrix on classification, we also performed statistical significance testing based on PAR values to compute effect sizes. These results are provided in the Supplementary Material to avoid extending this article further.

## Discussion

The effectiveness of applying Q-matrix-based CDMs to diagnostic assessments mainly depends on their statistical properties. One of the properties is statistical identifiability, which is the feasibility of recovering model parameters based on observed data. Identifiability is a prerequisite for model parameter estimation, which includes student class membership estimation. One of the sufficient and necessary conditions to guarantee identifiability when certain types of CDMs are utilized is the completeness of the Q matrix (Xu and Zhang, 2016; Xu, 2017). When attributes are independent, the concept of completeness of a Q matrix was first proposed by Chiu et al. (2009). Completeness means that a Q matrix can distinguish two different attribute vectors based on an ideal response pattern. The results of the above study indicate that the Q matrix must include an identity matrix to guarantee the separation of different ideal response patterns. This means that the diagnostic test must include items that only measure a single attribute to guarantee good classification results. However, in a situation where attributes are hieratically related to each other, where a certain attribute is a prerequisite for other attributes, including items that only measure a single attribute may not be feasible.

Identifiability is also discussed in the framework of knowledge space theory (KST) (Heller et al., 2015, 2016, 2017). There are two main differences between the above studies and our study: (1) our study focuses on cognitive diagnosis theory, whereas the above studies focused on KST; (2) our study is based on restricted Q matrix design. In many application studies (e.g., Tatsuoka, 1990, 2009; Leighton et al., 2004; Gierl, 2007, 2008; Wang and Gierl, 2011), Q matrix design was restricted. To address this concern, under the framework of CDMs, this study focused on identifiability based on unrestricted Q matrix design, where the test Q matrix represents a special attribute hierarchy structure (such as divergent, convergent, or linear structures) and if an item measures one attribute, then it should also measures all of its prerequisite attributes. Additionally, a simpler operational procedure for constructing an identifiable Q matrix was provided in a step-by-step manner to aid practitioners.

Based on the studies by Ding et al. (2008, 2012, 2016), we formally define a complete Q matrix design in a more general framework when an attribute hierarchy exits. The proposed complete Q matrix design can be easily constructed from an R matrix, which reflects the direct and indirect relationships between attributes. When attributes are independent, the proposed design is equivalent to that presented by Chiu et al. (2009). Such a Q matrix is an important condition for guaranteeing accurate classification results when combined with an attribute profile estimation approach utilizing conjunctive assumptions, such as the RSM, AHM, and a family of conjunctive restricted latent class models. The complete restricted Q matrix design is equivalent to the model identifiability condition for the DINA model when item parameters are known, which follows from the logic outlined by Xu and Zhang (2016).

Because completeness is only one of the important conditions for the identifiability of a Q-matrix-based CDM, a very important future research direction is to study additional conditions related to Q matrix design and discuss additional model identifiability conditions. Our current work only indicates that including one R matrix in the design can guarantee the identifiability of an ideal response pattern, which is one of the model identifiability conditions. Additional conditions must be investigated to guarantee the identifiability of attribute profiles and item parameters.

Another limitation of this study is that we only focused on conjunctive CDMs, which are naturally appropriate for the hierarchical attribute assumption. The impact of an ideal response pattern on classification accuracy is larger for conjunctive models than for compensatory models. We expect that completeness is not sufficient to guarantee good classification results in a compensatory model. It would be worth to investigating Q matrix design when utilizing general models, such as the log-linear cognitive diagnostic model (LCDM; Henson et al., 2009), the generalized deterministic input, noisy, “and” gate (G-DINA; De La Torre, 2011) model, and other general diagnostic models. Liu et al. (2016) conducted a simulation study to investigate three different Q matrix designs by utilizing the hierarchical log-liner model (Templin and Bradshaw, 2014). However, their Q matrix design seems to be a mixed structure, which contains both items that satisfy the specified attribute hierarchy and items that do not satisfy the specified hierarchy. In the future, it is worth investigating mixed-type Q matrix design.

The third limitation of this study is that it mainly focused one factor (i.e., attribute hierarchy) that affected identifiability. However, there are many other factors, such as the completeness and accuracy of the test Q matrix, number of attributes, examinees, items, item quality, and distribution of examinees, which may affect identifiability. To avoid excessive complexity, the test Q matrix was assumed to be correct to avoid the effects of an incorrect test Q matrix. The number of items and attributes were fixed as 30 and six, respectively, which are popular choices in real-world applications. Additionally, the sample size was fixed as 1,000. We expect that the effect size observed in simulations would decrease as the sample size decreases. However, in this study, because our tests were intended to measure six attributes and there are 2^6^ = 64 types of attribute profiles or categories, even when a large sample size of *N* = 1,000 was generated, there were only approximately 19 participants in each category, which is a marginal number for correctly evaluating categories. Furthermore, the distributions of items and examinee parameters were fixed and followed a common distribution to avoid the effects of item quality and population size. In the future, if a smaller number of attributes is measured, a smaller sample size may be investigated. Other factors may also be investigated to produce more general results.

## Author contributions

YC and SD design of the study, data analysis, paper writing, and revision. DT data analysis and interpretation of data.

### Conflict of interest statement

The authors declare that the research was conducted in the absence of any commercial or financial relationships that could be construed as a potential conflict of interest.
