# Supplementary material for: Theorems and Methods of a Complete Q Matrix With Attribute Hierarchies Under Restricted Q-Matrix Design
Source: Front Psychol. 2018 Aug 8;9:1413. doi: 10.3389/fpsyg.2018.01413 (PMC6092632; doi:10.3389/fpsyg.2018.01413)
Supplement: Supplementary file 1 [file Data_Sheet_1.DOCX]

**Supplementary Material:**

**Analysis on statistical testing on significance and its effect size**

To detect the effect size of pattern-wise agreement rate (PARs), the Z statistic testing on significance between the test Q matrix with R matrix and other test Q matrixes is done and the effect size (Cohen’s d) is also computed. The testing results of five models when one of the columns of the R matrix is missing in the test Q matrix are documented in below Table 1. We can observe low effect size (0.09~0.12) among 2/7 percent of experiment conditions. The results reflect that the influences of R matrix on PARs are different under different hierarchical structures and models. From the aspect of models, there has the strongest influence for AHM, and the next are DINA, RSM and NIDA model while having the weakest influence for the rRUM. From the aspect of attribute structures, there has the strongest influence for the convergent structure, and the next for Linear, independent and divergent structure, respectively.

The testing results of five models when the entire R matrix is missing from the test Q matrix are documented in Table 2. We can observe greater effect size (0.23~0.53) and obvious decrease of effect size across structures in this extreme case. Compared the effect size under independent structure with divergent structure, the decrease is -0.05 for RHM, 0.08 for AHM, 0.11 for DINA, 0.17 for NIDA and 0.11 for reduced RUM. Specifically, the influences of R matrix on PARs across models are similar, except that there has stronger influence for NIDA model than that for RSM.

**Table 1** Results of statistical testing on significance for five conjunctive models when one column of R matrix missing in the test Q matrix

| Structure | The missing item type | RSM | | AHM | | DINA | | NIDA |  | rRUM | |
| --- | --- | --- | --- | --- | --- | --- | --- | --- | --- | --- | --- |
|  |  | z | d | z | d | z | d | z | d | z | d |
| Linear | [100000]’ | 1.93 | 0.09 | 2.64^**^ | 0.12 | 2.34^*^ | 0.10 | 1.47 | 0.07 | 2.08^*^ | 0.09 |
|  | [110000]’ | 2.25^*^ | 0.10 | 2.61^**^ | 0.12 | 2.29^*^ | 0.10 | 1.24 | 0.06 | 1.25 | 0.06 |
|  | [111000]’ | 1.84 | 0.08 | 2.56^*^ | 0.11 | 2.31^*^ | 0.10 | 1.27 | 0.06 | 1.76 | 0.08 |
|  | [111100]’ | 1.15 | 0.05 | 2.56^*^ | 0.11 | 2.36^*^ | 0.11 | 1.59 | 0.07 | 1.53 | 0.07 |
|  | [111110]’ | 2.31^*^ | 0.10 | 2.51^*^ | 0.11 | 2.24^*^ | 0.10 | 1.74 | 0.08 | 1.72 | 0.08 |
|  | [111111]’ | 1.46 | 0.07 | 2.74^**^ | 0.12 | 2.39^*^ | 0.11 | 1.89 | 0.08 | 1.46 | 0.07 |
| Convergent | [100000]’ | 2.40^*^ | 0.11 | 2.09^*^ | 0.09 | 2.10^*^ | 0.09 | 1.26 | 0.06 | 0.95 | 0.04 |
|  | [110000]’ | 2.04^*^ | 0.09 | 1.99^*^ | 0.09 | 2.10^*^ | 0.09 | 0.56 | 0.03 | 0.74 | 0.03 |
|  | [111000]’ | 1.78 | 0.08 | 2.24^*^ | 0.10 | 2.45^*^ | 0.11 | 1.83 | 0.08 | 1.33 | 0.06 |
|  | [111100]’ | 2.13^*^ | 0.10 | 2.34^*^ | 0.10 | 2.17^*^ | 0.10 | 1.83 | 0.08 | 1.30 | 0.06 |
|  | [111110]’ | 2.01^*^ | 0.09 | 2.06^*^ | 0.09 | 2.02^*^ | 0.09 | 1.18 | 0.05 | 1.23 | 0.06 |
|  | [111111]’ | 1.81 | 0.08 | 2.21^*^ | 0.10 | 2.00^*^ | 0.09 | 1.61 | 0.07 | 2.52^*^ | 0.11 |
| Divergent | [100000]’ | 0.15 | 0.01 | 1.15 | 0.05 | 0.51 | 0.02 | 0.51 | 0.02 | 0.14 | 0.01 |
|  | [110000]’ | 0.69 | 0.03 | 1.59 | 0.07 | 1.40 | 0.06 | 1.16 | 0.05 | 0.55 | 0.02 |
|  | [111000]’ | 0.69 | 0.03 | 1.51 | 0.07 | 1.38 | 0.06 | 1.55 | 0.07 | 1.05 | 0.05 |
|  | [111100]’ | 1.29 | 0.06 | 1.59 | 0.07 | 1.43 | 0.06 | 0.48 | 0.02 | 0.33 | 0.01 |
|  | [111110]’ | 1.10 | 0.05 | 1.78 | 0.08 | 1.33 | 0.06 | 1.42 | 0.06 | 1.22 | 0.05 |
|  | [111111]’ | 1.00 | 0.04 | 1.59 | 0.07 | 1.59 | 0.07 | 1.21 | 0.05 | 1.02 | 0.05 |
| Independent | [100000]’ | 1.36 | 0.06 | 2.05^*^ | 0.09 | 0.78 | 0.03 | 2.54^*^ | 0.11 | 0.99 | 0.04 |
|  | [110000]’ | 1.04 | 0.05 | 1.89 | 0.08 | 1.70 | 0.08 | 1.50 | 0.07 | 1.01 | 0.05 |
|  | [111000]’ | 1.28 | 0.06 | 2.50^*^ | 0.11 | 1.25 | 0.06 | 2.13^*^ | 0.10 | 0.88 | 0.04 |
|  | [111100]’ | 0.88 | 0.04 | 2.10^*^ | 0.09 | 1.28 | 0.06 | 1.84 | 0.08 | 0.80 | 0.04 |
|  | [111110]’ | 1.20 | 0.05 | 2.28^*^ | 0.10 | 1.54 | 0.07 | 2.07^*^ | 0.09 | 1.01 | 0.05 |
|  | [111111]’ | 1.00 | 0.04 | 2.28^*^ | 0.10 | 0.96 | 0.04 | 1.73 | 0.08 | 1.15 | 0.05 |

*Note*: ^*^ and ^**^ indicate that the difference is significant at the probability level 0.05and 0.01 respectively. Z= Z statistic; d= Cohen’s d

**Table 2**  Results of statistical testing on significance for five conjunctive models when test Q matrix does not contain the R matrix

| Structure | RSM | | AHM | | DINA | | NIDA |  | rRUM | |
| --- | --- | --- | --- | --- | --- | --- | --- | --- | --- | --- |
|  | z | d | z | d | z | d | z | d | z | d |
| Independent | 6.42^***^ | 0.29 | 11.58^***^ | 0.52 | 11.26^***^ | 0.50 | 11.75^***^ | 0.53 | 7.60^***^ | 0.34 |
| Divergent | 7.71^***^ | 0.34 | 9.90^***^ | 0.44 | 8.79^***^ | 0.39 | 6.69^***^ | 0.30 | 5.22^***^ | 0.23 |

*Note*: ^***^ indicates that the difference is significant at the probability level of 0.001. Z= Z statistic; d= Cohen’s d
