# Supplementary material for: Theorems and Methods of a Complete Q Matrix With Attribute Hierarchies Under Restricted Q-Matrix Design
Source: Front Psychol. 2018 Aug 8;9:1413. doi: 10.3389/fpsyg.2018.01413 (PMC6092632; doi:10.3389/fpsyg.2018.01413)
Supplement: Supplementary file 2 [file Data_Sheet_2.docx]

**Appendix**

**1. An attribute hierarchical structure as a graph**

Graph Theory is a branch of mathematics that has been widely employed in connections with three diagrams consisting of nodes and arcs. Based on Graph Theory, Tatsuoka (1986) introduced Adjacent matrix and Reachability matrix to describe relationship between attributes, and Yang et al. (2008) discussed the important properties of Reachability matrix. To introduce some important results from Yang et al. (2008), we first discuss attribute hierarchies in the Graph Theory framework.

The attribute hierarchies refer to situations in which the mastery of a certain attribute is a prerequisite to the mastery of another attribute. Figure 1 presents four types of comment attribute hierarchies introduced by Leighton et al. (2004). Let S denote an attribute hierarchy, then S can regard a graph with attributes in the structure as vertices or nodes. For example, if S denote the linear attribute structure(A) in Figure 1, the vertex set of S can be written as $V\left(S\right)=\left\{A_{1},A_{2},A_{3},A_{4},A_{5},A_{6}\right\}$, where$A_{i}$represents the attribute i. Hereafter, the term “vertex” is equivalent to “attribute”. If vertex x is the direct or indirect prerequisite for vertex y, then x is a prerequisite vertex of vertex y. A vertex is named as a start vertex, if it does not have any prerequisite vertices. A vertex is defined as a leaf vertex, if it is not the prerequisite vertex of any other vertices. For example, if S denotes the divergent structure (C) in Figure 1, then A1 is the start vertex, A3, A5 and A6 are leaf vertices. A1 is the prerequisite vertex of A2, A1 and A2 are prerequisite vertex of A3. Based on the above definitions, it’s easy to get the following two properties (Yang et al. (2008)):

**A.1** In a hierarchical structure S, for a node x, define$P_{x}$={$y\in S$, y is a prerequisite vertex of x}. If$x_{1}$is the prerequisite vertex of$x_{2}$, then$P_{x_{1}}\subset P_{x_{2}}$. In another word, the prerequisite vertices of $x_{1}$ are also the prerequisite vertices of $x_{2}$.

**A.2** Suppose S is an attribute hierarchical structure, and I is an item which satisfies such structure. Then I can regard as a subgraph S_1_ of S. Let V (S_1_) be the vertex set of S_1_, then $\forall x\in S_{1}$, let $V'$= { y | y is the prerequisite vertex for x }, then $V'$⊂ V (S_1_).

Next several important results from Yang et al. (2008) will be introduced, which will support our complete Q matrix theory in section 3.

**2. Important results from Yang et al. (2008)**

All the lemmas and Theorems in this section were from Yang et al. (2008). For the convenience of readers to refer their results, we reorganize and restate them as follows.

**Lemma 1.** Suppose S_1_ is a subgraph of graph S which represents an attribute structure, then S_1_ represents an item which satisfies the specified structure in S, if and only if $\cup_{x\in S_{i}}P_{x_{1}}\subset S_{1}$.

Lemma 1 can be easily proofed by using **A.2** and the definition of the attribute hierarchies.

**Lemma 2.** Suppose S represents an attribute hierarchical structure, and R is the corresponding reachability matrix. Then each column of R represents an item which satisfied S．

Proof. This Lemma is the Proposition 1 presented in current manuscript. Based on the set up for the R matrix in the current manuscript, if there are K attributes, and we label the attributes from the highest level to the lowest level with an ascending sequence from 1 to K, then the R matrix is an upper-triangular matrix with diagonals as 1s. According to the definition of the R matrix in the this manuscript, for the *jth* column of the R matrix, $\mathbf{r}_{\mathbf{j}}=\left(r_{1j},\cdots,r_{jj},\cdots,r_{Kj}\right)^{'}$, for $k<j$,$r_{kj}=1$ if A_k_ is the prerequisite vertex of A_j_; for $k=j$, $r_{jj}=1$; for $k>j$,$r_{jk}=0$. Let $S_{j}$be the corresponding graph representation of $\mathbf{r}_{j}$, then the vertex k contained in S_1_ are the attribute k such that $r_{kj}=1$. If we define $P_{A_{j}}$= {A_k_ |A_k_ is the prerequisite vertex of A_j_}, then $P_{A_{j}}\subset S_{j}$. Then based on property **A.1**, we can get that $\cup_{x\in S_{2}}P_{x}=P_{A_{j}}$. Using the result from Lemma 1, we complete the proof for Lemma 2.

**Theorem 3.** Let S represents a hierarchical structure for K attributes, R is the corresponding reachability matrix, and $R=\left(\mathbf{r}_{\mathbf{1}},\cdots,\mathbf{r}_{\mathbf{K}}\right)$. Let $\mathbf{I}=\left(b_{1},\cdots,b_{K}\right)^{'}$,$b_{k}\in\left\{0,1\right\}$,

$k\in\left\{1,2,\cdots,K\right\}$is an item which satisfies S. Then

$$\mathbf{I}=b_{1}\times\mathbf{r}_{\mathbf{1}}+b_{2}\times\mathbf{r}_{\mathbf{2}}+...+b_{K}\mathbf{r}_{K}=\sum_{k=1}^{K} b_{k}\mathbf{r}_{k}$$

Proof. Let S_1_ be a subgraph of S which represents item **I**. For any k such that$b_{k}=1$, the $\mathbf{r}_{k}$ in R represents all the prerequisite vertices of A_k_ (including A_k_ itself), and they all belong to S_1_ because **I** is an item which statistics S. Then the summation $\sum_{k=1}^{K} b_{k}r_{k}$can represent a set denote I_p_ that contains all prerequisite vertices of vertices in S_1_. Then according to Boolean addition “ + ”, I_p_ ⊂ S_1_. Then based on Lemma 1, S_1_ ⊂ I_p_. This concludes S_1_ = I_p_, that is$\mathbf{I}=\sum_{k=1}^{K} b_{k}\mathbf{r}_{k}$.

Remark 2. Note that this theorem is equivalent to the Proposition 2 in the current manuscript, because each column of *Qr* can regard an item which satisfies S.

**Theorem 4**. Let S represents a hieratical structure for K attributes, and R is the corresponding reachability matrix. Let SR represents the matrix derived from Augment algorithm corresponding to R matrix. Then the columns of the SR matrix are equivalent to all item types that are satisfied the structure S.

Proof. Let’s first define$R=\left(\mathbf{r}_{1},\cdots,\mathbf{r}_{K}\right)$, and let $\mathbf{I}=\left(b_{1},\cdots,b_{K}\right)^{'}$, $b_{k}\in\left\{0,1\right\}$be an item that satisfies S. According to Theorem 3, $\mathbf{I}=\sum_{k=1}^{K} b_{k}\mathbf{r}_{k}=\sum_{k\in\left\{\left.k\right|b_{k}=1\right\}} r_{k}$. If **I** belongs to the item types defined in the R matrix, then we are done with the proof. Otherwise, suppose the cardinality of $k\in\left\{\left.k\right|b_{k}=1\right\}=n\geq2$, and we denote the columns corresponding to those attributes as $\left(r_{\mathbf{I}_{1}},\cdots,r_{\mathbf{I}_{n}}\right)$. To simplify the argument, we assume the subscript of $r_{\mathbf{I}_{k}},k\in\left\{1,2,\cdots,n\right\}$keep the same positions as those in the original R matrix. That is **I**_k_ represents the **I**_k_th column in the R matrix. Then based on the Augment algorithm, during at most the In the iteration, we will be able to obtain an item which can be represented as $\sum_{k=1}^{n} r_{\mathbf{I}_{k}}$. The proof is completed.

1. **Proof for the main theorems**

Proof for Theorem 1

Proof. Suppose R matrix is not complete, then there exists at least two attribute patterns, say$\mathbf{\alpha}^{1},\mathbf{\alpha}^{2}\in M$ , such that$\mathbf{\alpha}^{1}\neq\mathbf{\alpha}^{2}$, but $\eta\left(R,\mathbf{\alpha}^{1}\right)=\eta\left(R,\mathbf{\alpha}^{2}\right)$.

**Case 1**: If one of the two attribute patterns is a zero vector, say$\mathbf{\alpha}^{1}=\mathbf{0}_{K}$, then we can get $\eta\left(R,\mathbf{\alpha}^{1}\right)=\mathbf{0}_{K}$. Because$\eta\left(R,\mathbf{\alpha}^{1}\right)=\eta\left(R,\mathbf{\alpha}^{2}\right)$and the former assumption, then$\eta\left(R,\mathbf{\alpha}^{2}\right)=\mathbf{0}_{K}$. Because $\mathbf{\alpha}^{2}$is not a zero vector, then$\mathbf{\alpha}^{2}$belongs to one the column vector of the *Qr* matrix based on the relationship between M matrix and the *Qr* matrix illustrated in section 3.2. Regarding Proposition 3, we can conclude that there exists l ≥ 1 columns in R such that the attributes measured by each of those columns is a subset of the attributes measured by$\mathbf{\alpha}^{2}$. Let L denote the set containing the indexes of those columns, then$\mathbf{\alpha}^{2}=\sum_{i\in L} \mathbf{r}_{i}$, where $\mathbf{r}_{i}=\left(r_{1i},\cdots,r_{Ki}\right)^{'}$. Thus, for any $i\in L$, $\eta_{i}\left(R,\mathbf{\alpha}^{2}\right)=\prod_{k=1}^{K} \left(\mathbf{\alpha}_{k}^{2}\right)^{r_{ki}}=1$. This is contradictory to that$\eta\left(R,\mathbf{\alpha}^{2}\right)=\mathbf{0}_{K}$.

**Case 2:** If both $\mathbf{\alpha}^{1}$ and $\mathbf{\alpha}^{2}$ are non-zero vectors, then they belong to the columns of the *Qr* matrix. Based on Proposition 3, we have$\mathbf{\alpha}^{1}=\sum_{i\in L_{1}} \mathbf{r}_{i}$and$\mathbf{\alpha}^{2}=\sum_{i\in L_{2}} \mathbf{r}_{i}$, where$L_{1},L_{2}$denote the set containing the column indexes from the R matrix. Because$\mathbf{\alpha}^{1}\neq\mathbf{\alpha}^{2}$, we assume$\alpha_{k}^{1}=0$and$\alpha_{k}^{2}=1$ for generality. Because$\mathbf{\alpha}^{1}$is a linear combination of$\mathbf{r}_{i}$for$i\in L_{1}$, then there must exist one vector, say$\mathbf{r}_{e}$, such that $\left\{m\left|r_{me}=1\right.\right\}\subset\left\{m\left|\alpha_{m}^{2}=1\right.\right\}$and$r_{ke}=0$. Similarly, because$\mathbf{\alpha}^{2}$is a linear combination of$\mathbf{r}_{i}$,$i\in L_{2}$, then there must exist one vector, say $\mathbf{r}_{s}$, such that$r_{ks}=1$and$\left\{m\left|r_{ms}=1\right.\right\}\subset\left\{m\left|\alpha_{m}^{2}=1\right.\right\}$. Based on the above arguments, we can get that

$\eta_{e}\left(R,\mathbf{\alpha}^{1}\right)=1,\eta_{e}\left(R,\mathbf{\alpha}^{2}\right)=0,\eta_{s}\left(R,\mathbf{\alpha}^{1}\right)=0,\eta_{s}\left(R,\mathbf{\alpha}^{2}\right)=1$.

This is contradictory to$\eta\left(R,\mathbf{\alpha}^{1}\right)=\eta\left(R,\mathbf{\alpha}^{2}\right)$.

**Proof of Theorem 2**

Proof. **Proof of the Sufficiency**: Suppose R matrix is a sub-matrix of *Q_c_*. For simplicity, we first reorder the columns in *Q_c_* so that the R matrix becomes the first K columns in the test Q matrix. Then for any$\mathbf{\alpha}\in M$, $\eta\left(R,\mathbf{\alpha}\right)$is equivalent to the first K elements of$\eta\left(Q_{c},\mathbf{\alpha}\right)$, i.e.$\eta_{1:K}\left(Q_{c},\mathbf{\alpha}\right)$. According to Theorem 1, for any two permissible attribute patterns$\mathbf{\alpha}^{1},\mathbf{\alpha}^{2}\in M$, if$\mathbf{\alpha}^{1}\neq\mathbf{\alpha}^{2}$, we have$\eta\left(R,\mathbf{\alpha}^{1}\right)\neq\eta\left(R,\mathbf{\alpha}^{2}\right)$, and this implies that $\eta\left(Q_{c},\mathbf{\alpha}^{1}\right)\neq\eta\left(Q_{c},\mathbf{\alpha}^{2}\right)$, which concludes the sufficiency.

**Proof of the Necessity**: Suppose the test Q matrix *Q_c_* is complete, then for any two different permissible attribute patterns$\mathbf{\alpha}^{1},\mathbf{\alpha}^{2}\in M$, $\eta\left(Q_{c},\mathbf{\alpha}^{1}\right)\neq\eta\left(Q_{c},\mathbf{\alpha}^{2}\right)$. If R matrix is not a sub-matrix of *Q_c_*, then there must exist one column vector from R, say$\mathbf{r}_{e}=\left(r_{1e},\cdots,r_{Ke}\right)$,$e\in\left\{1,2,\cdots,K\right\}$, not belongs to the columns of *Q_c_*.

(1) If there only exists one non-zero element in **r**, then $r_{ee}=1$ and $r_{ek}=0$ for any $k\neq e$ based on the definition of R matrix. Then there exists two permissible attribute patterns $\mathbf{\alpha}^{1}=\mathbf{r}_{e}$ and $\mathbf{\alpha}^{2}=\mathbf{0}_{K}$, which satisfy $\mathbf{\alpha}^{1}\neq\mathbf{\alpha}^{2}$ and$\eta\left(Q_{c},\mathbf{\alpha}^{1}\right)=\eta\left(Q_{c},\mathbf{\alpha}^{2}\right)=\mathbf{0}_{K}$. This contradicts to the completeness of *Q_c_*.

(2) If there is at least two non-zero elements in **r**_e_, because R matrix is an upper triangular matrix, then $r_{ee}=1$ and $r_{ie}=1$ for some$i<e$. Define$P=\left\{i|r_{i\text{e}}=1,i\neq e\right\}$, then P contains all the prerequisites for attribute e. Based on the properties of R matrix, there exists an permissible attribute pattern$\mathbf{\alpha}^{1}$, such that$\alpha_{k}^{1}=0$**,** $k\notin P\cup\left\{e\right\}$and $\alpha_{k}^{1}=1$, $k\in P$. Based on the relationship between R matrix, *Qr* matrix and M matrix, $\mathbf{r}_{e}$can be treated as an permissible attribute pattern. Thus, let $\mathbf{\alpha}^{2}=\mathbf{r}_{e}$. Then $\mathbf{\alpha}^{1}$ and $\mathbf{\alpha}^{2}$ only differ in the e_th_ element, e.g. $\alpha_{e}^{1}=0$and$\alpha_{e}^{2}=1$. Suppose *Q_c_* is K ×J matrix, then$\eta_{j}\left(Q_{c},\mathbf{\alpha}^{1}\right)=\prod_{k} \left(\alpha_{k}^{1}\right)^{q_{kj}}$ and $\eta_{j}\left(Q_{c},\mathbf{\alpha}^{2}\right)=\prod_{k} \left(\alpha_{k}^{2}\right)^{q_{kj}}$ for any$j\in\left\{1,2,\cdots,J\right\}$.

K　　　　　　　 k

Let $S_{j}=\left\{k|q_{kj}=1\right\}$, then $S_{j}$contains all the attributes measured by the$j_{th}$item. Because **r**_e_ is not belongs to the columns of *Q_c_*, $S_{j}$satisfies that$S_{j}\neq P\cup\left\{e\right\}$. If $S_{j}-\left(S_{j}\cap P\cup\left\{e\right\}\right)=\emptyset$, which means all measured attributes have been mastered by people with the permissible attribute patterns$\mathbf{\alpha}^{1}$and$\mathbf{\alpha}^{2}$, then$\eta_{j}\left(Q_{c},\mathbf{\alpha}^{1}\right)=\eta_{j}\left(Q_{c},\mathbf{\alpha}^{2}\right)=1$. If $S_{j}-\left(S_{j}\cap P\cup\left\{e\right\}\right)\neq\emptyset$, which means there at least exists a measured attribute k which is not mastered by $\mathbf{\alpha}^{1}$ and$\mathbf{\alpha}^{2}$, then $\eta_{j}\left(Q_{c},\mathbf{\alpha}^{1}\right)=\eta_{j}\left(Q_{c},\mathbf{\alpha}^{2}\right)=0$. In summary, no matter in which case, we have for any j, $\eta_{j}\left(Q_{c},\mathbf{\alpha}^{1}\right)=\eta_{j}\left(Q_{c},\mathbf{\alpha}^{2}\right)$, and this contradicts to the completeness of *Q_c_*. This completes our proof for Theorem 2

1. **R matrices in the simulation study**

$$R_{linear}=\left(\begin{matrix} 111111 \\ 011111 \\ 001111 \\ 000111 \\ 000011 \\ 000001 \end{matrix}\right)$$

$$R_{convergent}=\left(\begin{matrix} 111111 \\ 011111 \\ 001011 \\ 000111 \\ 000011 \\ 000001 \end{matrix}\right)$$

$$R_{divergent}=\left(\begin{matrix} 111111 \\ 011000 \\ 001000 \\ 000111 \\ 000010 \\ 000001 \end{matrix}\right)$$

$$R_{independent}=\left(\begin{matrix} 100000 \\ 010000 \\ 001000 \\ 000100 \\ 000010 \\ 000001 \end{matrix}\right)$$
